# Bacterial and fungal diversities examined through high-throughput sequencing in response to lead contamination of tea garden soil

**DOI:** 10.3389/fmicb.2023.1121199

**Published:** 2023-03-22

**Authors:** Ziyan Zhang, Qingmei Deng, Hui Ye, Gaofei Ge

**Affiliations:** ^1^School of Resources and Environment, Anhui Agricultural University, Hefei, China; ^2^Biotechnology Centre, Anhui Agricultural University, Hefei, China

**Keywords:** multistage contamination, single instance contamination, bacterial diversity, fungal diversity, high-throughput sequencing, lead

## Abstract

Several studies have indicated that the heavy-metal content in tea is increasing gradually. Researchers examining the soil of more than 100 tea gardens in China have observed that lead content was higher in some soils. The effect of lead contamination on soil microorganisms in tea gardens was studied to determine the effect of lead on the essential functions of microorganisms in a tea garden soil ecosystem. Previous studies on pot experiments adopted the method of adding a single instance of pollution, which failed to comprehensively simulate the characteristics of the slow accumulation of heavy metals in soil. This study designed with two pollution modes (multistage and single instance) determined the content of soil lead in different forms according to the European Community Bureau of Reference extraction procedure. The community structure, species diversity and functional abundance of soil bacteria and fungi were examined by high-throughput sequencing. We observed that the content of four forms of lead was higher in the multistage contamination mode than in the single instance contamination mode. The effects of lead contamination on bacteria differed significantly (*p* < 0.05), and the abundance and diversity of bacteria were higher in the multistage contamination mode than in the single instance contamination mode. The community structure of fungi was more affected by lead than was that of bacteria. The content of each lead form was the environmental factor most strongly affecting soil bacteria and fungi. The predicted main function of the bacterial community was amino acid transport and metabolism, and the trophic mode of the fungal community was mainly pathotroph–saprotroph. This study revealed changes in soil microorganisms caused by different forms of lead and contamination methods in tea garden soil and provide a theoretical basis for examining the effects of lead contamination on soil microorganisms.

## 1. Introduction

Most heavy metals have long-term toxicological and other adverse effects on the environment and humans; thus, heavy-metal contamination has become a crucial environmental concern in numerous countries (Chen et al., 2018; Deng et al., 2018). Lead (Pb) is among the most common heavy-metal pollutants (Hou et al., 2019). Most Pb enters the environment in the form of “three wastes,” and only about 25% of Pb is recycled by humans. Pb can cause soil, water and air pollution, and because of its high toxicity, long residual time and easy concealment in soil, Pb contamination has attracted considerable attention from researchers in several countries. Studies have indicated that the content of heavy metals in tea is slowly increasing (De Oliveira et al., 2018; Zhang et al., 2018). Toxic heavy metals are absorbed through the food chain and drinking water (Wongsasuluk et al., 2014). Tea is among the most widely consumed beverages globally and has numerous health benefits (Bag et al., 2022), but Pb contamination in the soil of tea gardens has yet to be examined.

In the soil matrix, most Pb ions can form complexes with various organic and inorganic soil colloids, adsorb onto oxides and clays, and precipitate as carbonates, hydroxides and phosphates (Pourrut et al., 2011; Chen et al., 2015). Studies have indicated that the toxicity of heavy metals to microorganisms in soil is directly related to the bioavailability of heavy metals (Kot and Namiesnik, 2000; Wang et al., 2007). Sequential extraction procedures are widely used to determine the mobility and bioavailability of elements (Alan and Kara, 2019). These procedures could distinguish the mobile components from the residual components, thus characterizing the labile fractions (Tessier et al., 1979; Ure et al., 1995). However, each method often yielded different results. The European Community Reference Bureau (BCR) proposed a three-step sequential extraction procedure in 1992 (Ure et al., 1993), which Ettler (2016) and Li et al. (2020) then used to determine heavy-metal content.

Microorganisms regulate the biogeochemical processes in soil and thereby ensure its fertility and health (Aguilar-Paredes et al., 2020). These processes include decomposition, nutrient cycling, organic matter maintenance, pathogen control and pollutant degradation, which directly affect environmental quality (Mendes et al., 2013; Philippot et al., 2013; Bardgett and van der Putten, 2014). In topsoil ecosystems, bacteria and fungi typically account for more than 90% of total soil microbial biomass and are key regulators of organic matter dynamics and nutrient availability in soil (Chen et al., 2014). The accumulation of heavy metals in soil reduces microbial abundance, diversity and activity, leading to widespread environmental degradation (Gao et al., 2010; Yu et al., 2020). Jones and Lennon (2010) reported that microorganisms naturally resist metal toxicity through dormancy until the restoration of favorable conditions. The fungal groups in soil, including saprotrophic, symbiotrophic and pathotrophic fungi (Tedersoo et al., 2016), play central roles in the decomposition of organic matter and nutrient cycling (Uroz et al., 2016). Heavy metals in soil can affect the ability of fungi to perform their functions, leading to changes in the structure of fungal communities (Xie et al., 2016).

High-throughput sequencing is an effective method of determining the structure of a microbial community (Guo et al., 2017). It is often used to identify the structure of the microbial community in soil (Guo et al., 2017; Jia et al., 2020). Chen et al. (2021) used high-throughput sequencing to examine the metabolic potential and community structure of bacteria in tea garden soil.

Although studies have investigated the migration of Pb in tea garden soil and tea leaves, few, if any, studies have explored the effects of Pb on the microorganisms in tea garden soil. In addition, pot experiments on Pb-contaminated soil have all involved a single instance addition of the contaminant, which does not imitate the slow accumulation of low doses of heavy metals in soil. Moreover, few pot studies have examined heavy-metal contamination in tea plants in China, and most have examined tea garden soil without considering the effects of plants. This study investigated the effects of Pb contamination on the microbial activity and diversity of tea garden soil by adding the contaminant, Pb, at three concentrations and in several stages. The 16S rRNA and the ITS, respectively, performed on Pb-treated soil to explore the adaptive mechanism of the resident microbial communities. We hypothesized that the addition of the contaminant at several time points would be more conducive to elucidating its actual effects on the soil than would a single instance addition.

## 2. Materials and methods

### 2.1. Experimental materials and design

The experimental soil was collected from the Dabie Mountains in Anhui Province, China. After air drying, we removed impurities from the soil by using a 2-mm nylon sieve. The Pb content of the soil was 9.84 mg kg^−1^, soil pH was 3.93, soil moisture content was 3.03%, soil organic matter value was 2.29%, and the soil C/N value was 17.54. The test reagent was PbCl_2_, a standard reagent purchased from Aladdin Industrial Corporation. The pots used had a bottom diameter of 16 cm, a top diameter of 19 cm, and a height of 20 cm. Seedings of Shucha Zao, an annual cultivar of *Camellia sinensis* (tea) with a height of approximately 20 cm, subjected to Pb stress during the pot experiment. Tea seedlings were planted in the greenhouse from September to December 2020. During the planting process, the temperature was controlled at 20°C in the evening and 26°C in the day. During the experiment, Watered the soil once every 2 days, soil moisture concentration was regularly adjusted to 50% of total water holding capacity (WHC) with deionized water using weighing method.

Two modes of Pb contamination and three gradients of Pb concentration were used. The multistage Pb contamination mode comprised the following treatments: ML = 100 mg kg^−1^, MM = 300 mg kg^−1^ and MH = 900 mg kg^−1^. The single instance Pb contamination mode comprised the following treatments: OL = 100 mg kg^−1^, OM = 300 mg kg^−1^ and OH = 900 mg kg^−1^. No Pb contamination was used in the control treatment (CK). ML and OL represented low-concentration treatments of Pb, MM and OM represented medium-concentration treatments, and MH and OH represented high-concentration treatment. All experiments were repeated three times. For the single instance Pb contamination mode, the PbCl_2_ reagent was added to soil and mixed thoroughly to distribute it evenly, and the soil was equilibrated at room temperature for 1 week. Four tea seedlings were then planted in each pot and grown for 100 days in a greenhouse at Anhui Agricultural University. The accumulation of the contaminant was simulated by dividing the PbCl_2_ reagent into 10 portions on average and applying it to the soil every 10 days, for a total of 10 applications. The method of multistage application of PbCl_2_ to the tea pot soil was as follows: Pb pollutants were divided into 10 for each concentration gradient. The PbCl_2_ solid reagent was dissolved in deionized water and added to soil in the ML treatment at 10-day intervals with a Pb concentration of 10 mg kg^−1^, and the final concentration of Pb in ML treated soil was 100 mg kg^−1^ after 100 days. In this way, MM treatment added PbCl_2_ at 10-day intervals with a Pb concentration of 30 mg kg^−1^, MH treatment added PbCl_2_ at 10-day intervals with a Pb concentration of 90 mg kg^−1^ and the final concentration of Pb in MM and MH treatment soil was 300 and 900 mg kg^−1^ after 100 days, respectively. At the end of tea cultivation, the soil was collected from the pots for analysis.

### 2.2. Methods

Four forms of Pb in the soil were obtained using BCR extraction (Ure et al., 1993). The total content of each form was determined through inductively coupled plasma atomic emission spectroscopy. Total carbon and nitrogen content was measured through atomic absorption spectrophotometry, and the ratio of carbon to nitrogen (C/N) was calculated. Organic carbon content was determined using low-temperature potassium dichromate oxidation photocolorimetric method (Lu, 2000). Soil moisture was determined through weighting (Lu, 2000), and soil pH was determined using the potentiometric method, with a soil-to-water ratio of 2.5:1 (Lu, 2000).

Total genomic DNA was extracted using the soil DNeasy PowerSoil Pro Kit. The 16S rRNA amplicon sequencing and internal transcribed spacer (ITS1) amplicon sequencing were performed by Shanghai Tianhao Biotechnology. The integrity of genomic DNA was determined through agarose gel electrophoresis, and its concentration and purity were determined using NanoDrop 2000 and Qubit 3.0 spectrophotometers, respectively. Primers 341F (5′-CCTACGGGNGGCWGCAG-3′) and 805R (5′-GACTACHVGGGTATCTAATCC-3′) were used to amplify the V3 and V4 hypervariable regions of the bacterial 16S rRNA gene. The primers ITS1 (5′-CTTGGTCATTTAGAGGAAGTAA-3′) and ITS1 (5′-GCTGCGTTCTTCATCGATGC-3′) were used to strengthen the hypervariable region of the fungal *ITS1* gene. Sequencing was performed using the Illumina NovaSeq 6000 sequencer.

### 2.3. Statistical analysis

All data were processed using Excel 2016 and analyzed using SPSS 25.0. The high-throughput sequencing data were analyzed using the cloud platform of Shanghai Tianhao Biotechnology.

## 3. Results

### 3.1. Physical and chemical properties of soil

Table 1 lists the content of the four forms of Pb in the soil, which differed significantly among treatments (*p* < 0.05). Residual Pb content was the highest, followed by reducible and acid-soluble Pb, and oxidisable Pb was the lowest. The contents of the four forms of Pb were higher after multistage contamination than after single instance contamination. However, this difference was nonsignificant for low concentrations of Pb treatments (ML and OL) (*p* > 0.05); Pb content significantly differed between the medium-concentration Pb treatments (MM and OM), and between the high-concentration Pb treatments (MH and OH) (*p* < 0.05).

**Table 1:** Content of Pb forms in tea garden soil.

Treatments	Content of Pb in different forms (mg·kg^−1^)			
	Acid soluble Pb	Reducible Pb	Oxidisable Pb	Residual Pb
CK	0.04 ± 0.06 f	5.66 ± 0.28 e	0.40 ± 0.11 e	10.98 ± 1.63 e
ML	12.23 ± 0.59 e	42.73 ± 1.25 d	1.94 ± 0.35 d	48.50 ± 5.03 d
MM	57.49 ± 2.60 c	106.71 ± 4.38 c	5.87 ± 0.40 b	134.98 ± 13.29 d
MH	231.59 ± 6.63 a	249.69 ± 8.12 a	14.03 ± 1.41 a	411.65 ± 57.35 a
OL	10.22 ± 0.16 e	42.76 ± 0.52 d	2.12 ± 0.18 d	40.81 ± 1.87 d
OM	45.84 ± 0.52 d	102.97 ± 3.21 c	4.85 ± 0.31 c	123.19 ± 6.15 c
OH	189.29 ± 10.99 b	240.15 ± 12.59 b	13.33 ± 1.00 a	333.98 ± 19.54 b

Data are presented as the mean ± standard deviation. The lowercase letters represent significant differences (*p* < 0.05) among treatments.

Total Pb content did not significantly differ between the contamination methods for the ML and OL treatments (*p* < 0.05). For the medium- and high-concentration treatments, total Pb content was significantly higher after multistage contamination than after single instance contamination (*p* < 0.05). Total Pb content was highest in the MH treatment and lowest in the control treatment. Soil pH gradually decreased as Pb concentration increased in both contamination modes (Table 2). The pH was higher after single instance contamination than after multistage contamination for low and high Pb concentrations. However, the pH was higher after multistage contamination than after single instance contamination for medium Pb concentrations. The soil pH was the lowest in the MH treatment and highest in the control treatment. Water content was higher in the Pb-treated soil than in the control soil, and the effects of contamination mode and Pb concentration on water content were nonsignificant (*p* > 0.05). Water content increased with Pb concentration in both contamination modes (Table 2) and was higher after single instance contamination than after multistage contamination for low and high Pb concentrations. However, water content was higher after single instance contamination than after multistage contamination for the medium-concentration Pb treatment. Water content was the highest in the MH treatment and lowest in the control treatment.

**Table 2:** Physical and chemical properties of Pb-contaminated tea garden soil.

Treatments	Total Pb (mg·kg^−1^)	pH	Moisture content (%)	Organic matter (%)	C/N
CK	17.08 ± 1.72 f	3.60 ± 0.08 a	14.17 ± 0.67 c	2.33 ± 0.04 a	18.00 ± 3.88 a
ML	105.40 ± 3.54 e	3.49 ± 0.03 bc	16.66 ± 1.30 b	2.36 ± 0.04 a	15.26 ± 1.15 c
MM	305.04 ± 10.60 c	3.50 ± 0.08 bc	15.91 ± 0.95 b	2.28 ± 0.02 ab	13.99 ± 0.78 bc
MH	906.95 ± 62.59 a	3.44 ± 0.05 c	18.19 ± 0.84 a	2.35 ± 0.02 a	13.83 ± 1.18 bc
OL	95.90 ± 1.76 e	3.54 ± 0.02 ab	14.77 ± 1.03 c	2.22 ± 0.05 b	14.93 ± 0.87 bc
OM	276.85 ± 8.29 d	3.49 ± 0.02 bc	16.61 ± 0.57 b	2.19 ± 0.04 b	13.56 ± 0.94 bc
OH	776.75 ± 32.65 b	3.47 ± 0.03 bc	16.51 ± 1.14 b	2.23 ± 0.02 b	12.90 ± 0.61 c

Data are presented as the mean ± standard deviation. The lowercase letters represent significant differences (*p* < 0.05) among treatments.

The effects of Pb contamination on the organic matter in the soil significantly differed between contamination modes (*p* < 0.05). Organic matter content was significantly higher after multistage contamination than after single instance contamination (Table 2), and the difference in the effects of Pb concentration on organic matter content in the same contamination mode were nonsignificant (*p* > 0.05). Organic matter content was high with low and high Pb concentrations and low with medium Pb concentrations. After multistage contamination, organic matter content was higher in low and high Pb concentration treatments than in the control treatment. Pb contamination significantly reduced the C/N ratio (*p* < 0.05), which decreased as Pb concentration increased (Table 2). The C/N ratios were higher after multistage contamination than after single instance contamination under the same concentration conditions.

### 3.2. Operational taxonomic unit Venn diagram of soil microorganisms

**Figure 1:**
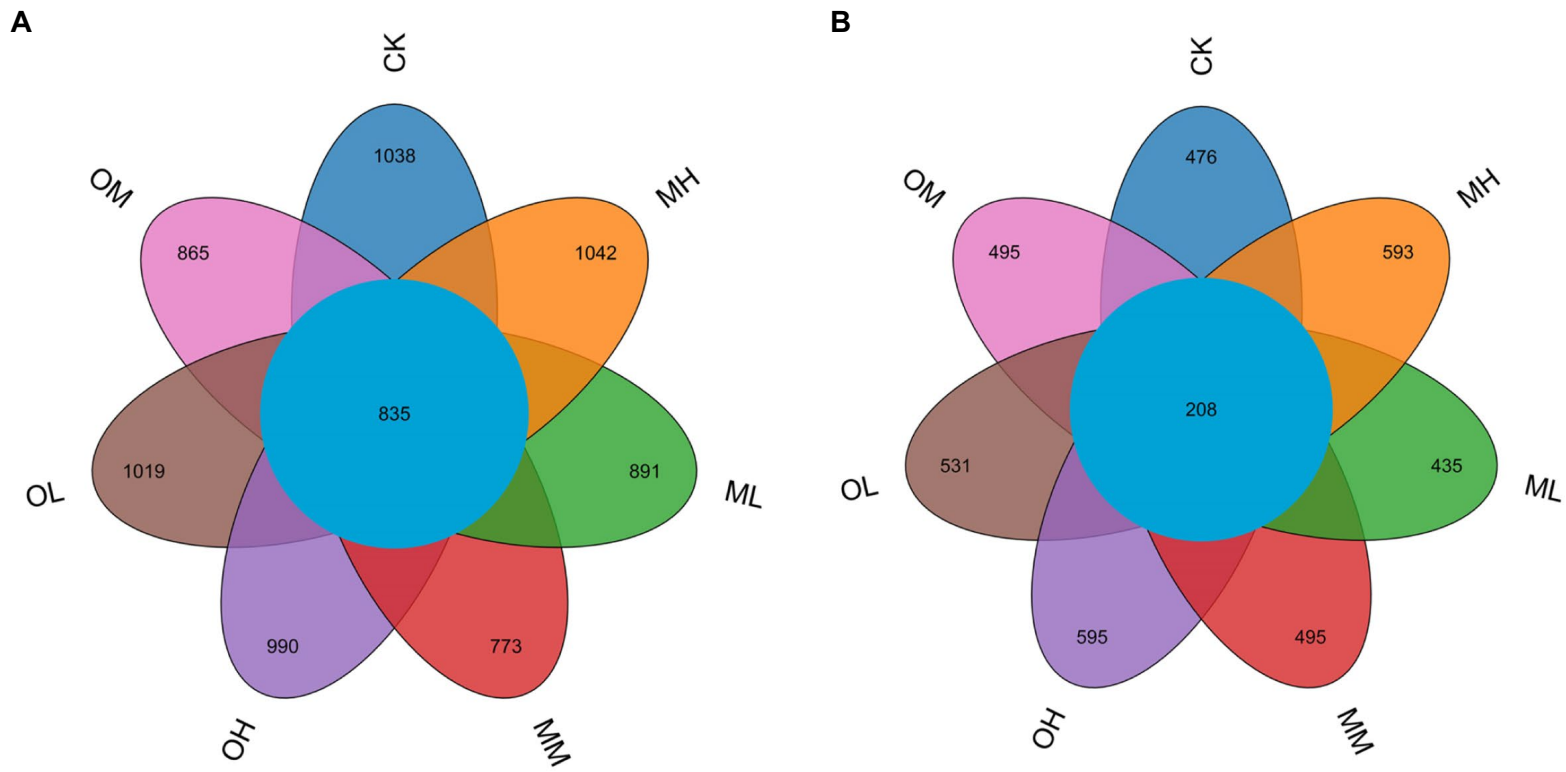
OTU Venn diagram of microorganisms in Pb-contaminated tea garden soil. **(A)** Bacteria, **(B)** Fungi.

We analyzed differences in bacterial operational taxonomic unit (OTU) numbers between the soil samples (Figure 1A). A total of 9,509 bacterial OTUs were detected in the samples. The total number of OTUs in the soils from the CK, ML, MM, MH, OL, OM and OH treatments was 2,923, 2,806, 2,558, 2,963, 2,847, 2,776 and 2,760, respectively, and the number of shared OTUs among the samples was 835. The number of shared OTUs was 1,038, 891, 773, 1,042, 1,019, 865 and 990, respectively. The number of OTUs was highest after MH and lower after the Pb treatments than after the control treatment. For low and medium Pb concentrations, the total and specific OTU numbers for bacteria were higher after single instance contamination than after multistage contamination. For the high Pb concentrations, the total and specific OTU numbers for bacteria were higher after MH than after OH. The total and specific OTU numbers for bacteria were higher after multistage contamination than after single instance contamination. After multistage contamination, bacterial abundance and diversity were highest for high Pb concentrations, followed by those for low and medium Pb concentrations. After single instance contamination, bacterial abundance and diversity were highest for the low Pb concentrations, followed by those for medium and high concentrations.

We also analyzed differences in fungal OTU numbers between treatments (Figure 1B). A total of 4,659 fungal OTUs were detected in the samples. Total OTUs in the CK, ML, MM, MH, OL, OM and OH samples were 1,037, 960, 1,081, 1,244, 1,170, 1,066 and 1,210, respectively, and the number of shared OTUs was 208; the number of unique OTUs was 476, 435, 495, 593, 531, 495 and 595, respectively. The number of total and specific OTUs was highest in the MH and OH samples. For low and high Pb concentrations, the number of total and specific OTUs numbers for fungi was higher after single instance contamination than after multistage contamination. For medium concentrations, the number of total and specific OTUs for fungi was higher after multistage contamination than after single instance contamination. Under identical Pb treatment conditions, the number of specific OTUs for fungi was higher after single instance contamination than after multistage contamination and the control treatment. After multistage contamination, fungal abundance and diversity were highest for high concentrations, followed by those for medium and low concentrations. After single instance contamination, fungal abundance and diversity were highest for high concentrations, followed by those for low and medium concentrations.

### 3.3. Microbial diversity

Table 3 lists the alpha diversity index for the bacteria in the soil samples treated with each Pb concentration. These values indicate the abundance and diversity of bacteria in the soil. Coverage in the seven samples was more than 99.9%, which indicated near-perfect coverage. The Chao1 and abundance-based coverage estimator (ACE) indices indicated the abundance of bacteria widely used in ecology. Chao1 and ACE were higher in the OL and MH treatments than in the control treatment but lower in the other treatments than in the control treatment. Pb contamination in the OL and MH samples increased bacterial diversity. With low and medium Pb concentrations, Chao1 and ACE were higher after single instance contamination than after multistage contamination. However, with high Pb concentrations, Chao1 and ACE were higher after multistage contamination than after single instance contamination. The Shannon and Simpson indices indicated the diversity of bacteria. A higher Shannon index indicates higher diversity, whereas a higher Simpson index indicates lower bacterial diversity. The Shannon indices were higher in the MH and OM samples than in the control sample. However, the Simpson indices were lower in the MH and OM samples than in the control sample, indicating that bacterial diversities were higher in the MH and OM samples. With low and medium Pb concentrations, the Shannon indices were higher after single instance contamination than after multistage contamination, whereas the Simpson indices were lower after single instance contamination than after multistage contamination; the opposite pattern was noted for high concentrations. The results indicate that the abundance and diversity of bacteria were high in soils after single instance low- and medium-Pb concentration contamination and multistage high-Pb concentration contamination.

**Table 3:** Alpha diversity index of bacteria in Pb-contaminated tea garden soil.

Treatment	Chao1	ACE	Shannon	Simpson	Coverage (%)
CK	1,518 ± 66 ab	1,521 ± 66 ab	6.49 ± 0.09 a	0.0034 ± 0.0006 a	99.95
ML	1,492 ± 48 ab	1,491 ± 49 ab	6.46 ± 0.06 a	0.0035 ± 0.0004 a	99.94
MM	1,385 ± 124 b	1,387 ± 126 b	6.43 ± 0.11 a	0.0036 ± 0.0007 a	99.97
MH	1,560 ± 39 a	1,562 ± 41 a	6.55 ± 0.03 a	0.0031 ± 0.0003 a	99.96
OL	1,532 ± 120 ab	1,535 ± 122 ab	6.50 ± 0.09 a	0.0034 ± 0.0003 a	99.95
OM	1,481 ± 74 ab	1,484 ± 75 ab	6.53 ± 0.08 a	0.0031 ± 0.0005 a	99.96
OH	1,478 ± 58 ab	1,478 ± 59 ab	6.44 ± 0.07 a	0.0039 ± 0.0008 a	99.95

Data represent the average of three replicates ± standard deviations. The lowercase letters in each column indicate significant differences (*p* < 0.05) among the Pb treatments.

Table 4 lists the alpha diversity index values for fungi in the soils treated with each Pb concentration and indicates the abundance and diversity of fungi in the soil. The coverage of the seven samples was more than 99.9%. Chao1 and ACE were higher in the MH and OH samples, indicating that the abundances of fungi were higher in the soils treated with high Pb concentrations. For the low and medium Pb concentrations, the Shannon and Simpson indices were highest in the OL sample, followed by those in the ML and OM samples, and the lowest value was in the MM soil sample, indicating that the Shannon and Simpson indices were higher after single instance contamination than after multistage contamination. The Shannon and Simpson indices were higher in the MH soil sample than in the OH soil sample for high Pb concentrations, indicating that the Shannon and Simpson indices were higher after multistage contamination than after single instance contamination. These results indicate that the abundance and diversity of fungi in the soils treated with low and medium Pb concentrations were higher after single instance contamination than after multistage contamination; the opposite pattern was observed for diversity of fungi in the soils treated with high Pb concentrations. In the multistage contamination mode, the abundance and diversity of fungi were lower in the soils treated with low Pb concentrations than in the control soil. The abundance and diversity of soil fungi increased with Pb concentration. Pb contamination increased the abundance and diversity of soil fungi in the single instance contamination mode, and the increase was stronger under low- and high-Pb concentration than under medium-Pb concentration.

**Table 4:** Alpha diversity index of fungi in Pb-contaminated tea garden soil.

Treatment	Chao1	ACE	Shannon	Simpson	Coverage (%)
CK	471 ± 72 bc	472 ± 72 bc	3.994 ± 0.261 b	0.050 ± 0.014 a	99.996
ML	456 ± 48 c	456 ± 48 c	4.026 ± 0.190 b	0.042 ± 0.010 ab	99.999
MM	512 ± 20 abc	513 ± 20 abc	4.234 ± 0.116 ab	0.034 ± 0.005 bc	99.994
MH	583 ± 69 a	583 ± 69 a	4.465 ± 0.032 a	0.028 ± 0.002 c	99.998
OL	557 ± 50 abc	558 ± 50 abc	4.486 ± 0.057 a	0.025 ± 0.002 c	99.996
OM	508 ± 62 abc	509 ± 62 abc	4.309 ± 0.153 a	0.029 ± 0.004 c	99.997
OH	566 ± 43 ab	567 ± 43 ab	4.448 ± 0.072 a	0.027 ± 0.004 c	99.996

Data represent the average of three replicates ± standard deviations. The lowercase letters in each column indicate significant differences (*p* < 0.05) among the Pb treatments.

**Figure 2:**
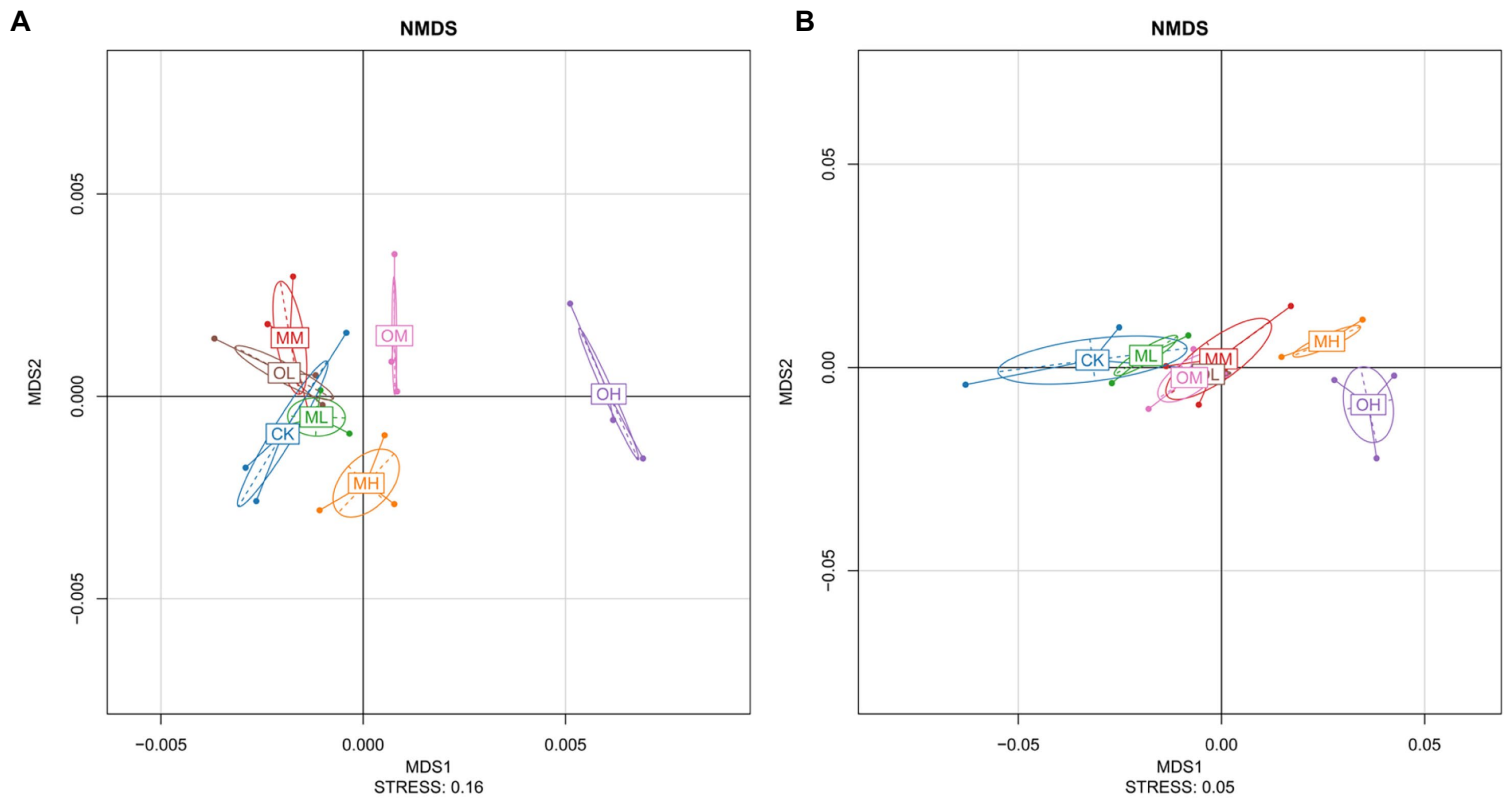
NMDS Analysis of Pb-contaminated tea garden soil. **(A)** Bacteria, **(B)** fungi.

Figure 2A presents the results of the nonmetric multidimensional scaling (NMDS) analysis of bacteria, performed using the weighted unifrac algorithm and a stress value of 0.16. The farther the distance between different Pb treatments the greater the difference. The MH, OM and OH values were notably different and exhibited longer distances from those of the other treatments (Figure 2A). Considerable overlap among CK, MM, MM, and OL was observed. OL, OM, and OH were notably different, and ML, MM, and MH were highly distinguishable. The NMDS analysis revealed a considerable difference in bacteria among the single instance contamination mode, multistage contamination mode and control treatment, indicating that the Pb contamination modes resulted in considerable differences in bacteria. The Pb concentrations resulted in considerable differences in the single instance contamination mode and slight differences in the multistage contamination mode. These differences increased with Pb concentration, indicating that each concentration resulted in considerable differences in soil bacteria.

Figure 2B presents the results of the NMDS analysis for fungi, performed using the Wunifrac algorithm and a stress value of 0.05. MH and OH differed considerably. More overlap was observed between CK and ML than among OL, OM and MM. The higher the Pb concentration was, the greater the difference was between fungi in the soil treated with Pb and in the control soil. In the soils treated with low and high Pb concentrations, fungi considerably differed between multistage and single instance contamination modes. Significant differences in fungi were noted among the soils treated with different Pb concentrations in the multistage contamination mode (*p* < 0.05). In the single instance contamination mode, the difference in fungi was smaller between the soils treated with low and medium Pb concentrations and larger between the soils treated with high Pb concentrations.

### 3.4. Composition of soil microbial community

**Figure 3:**
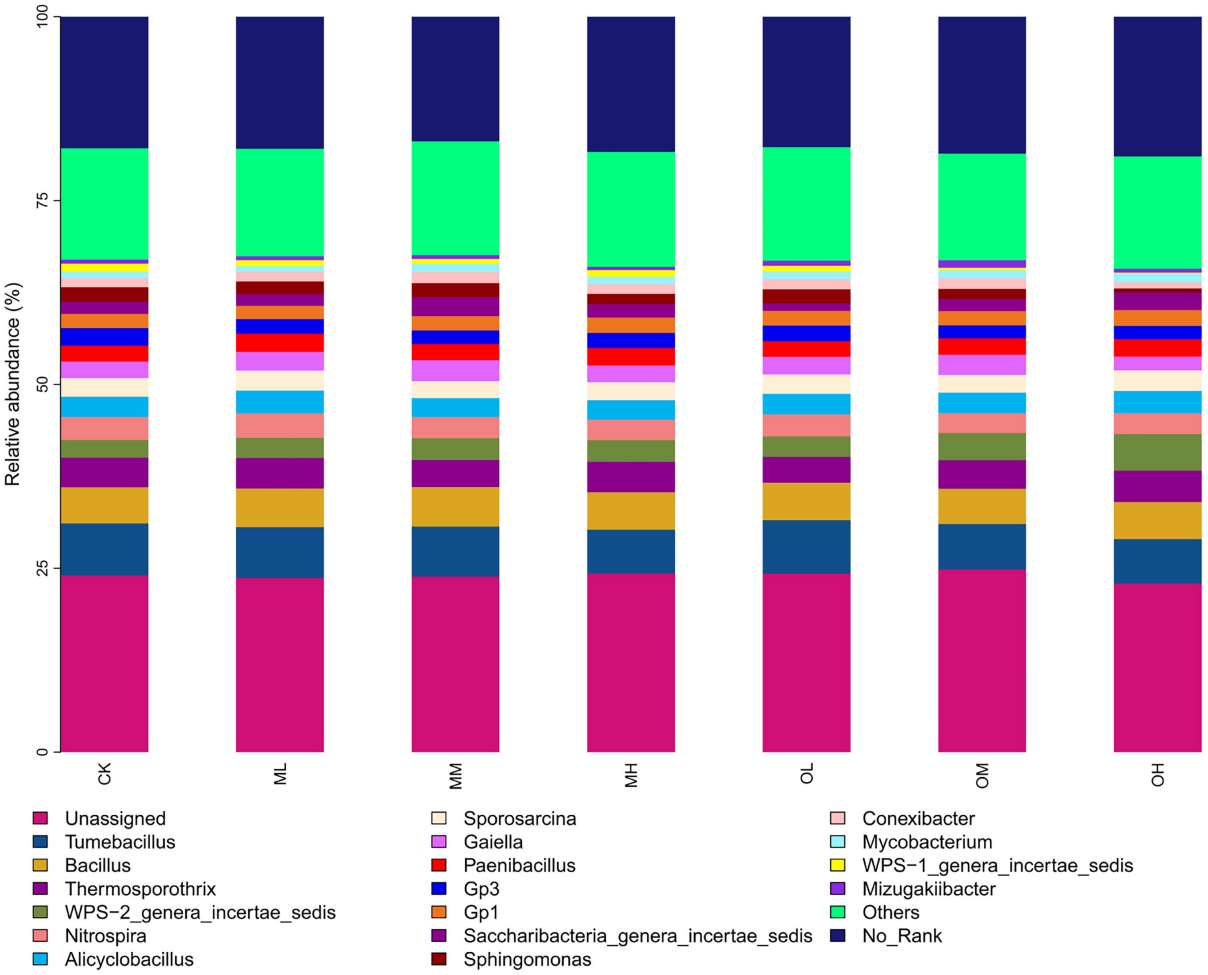
Bacterial genus-level species composition in Pb-contaminated tea garden soil.

**Figure 4:**
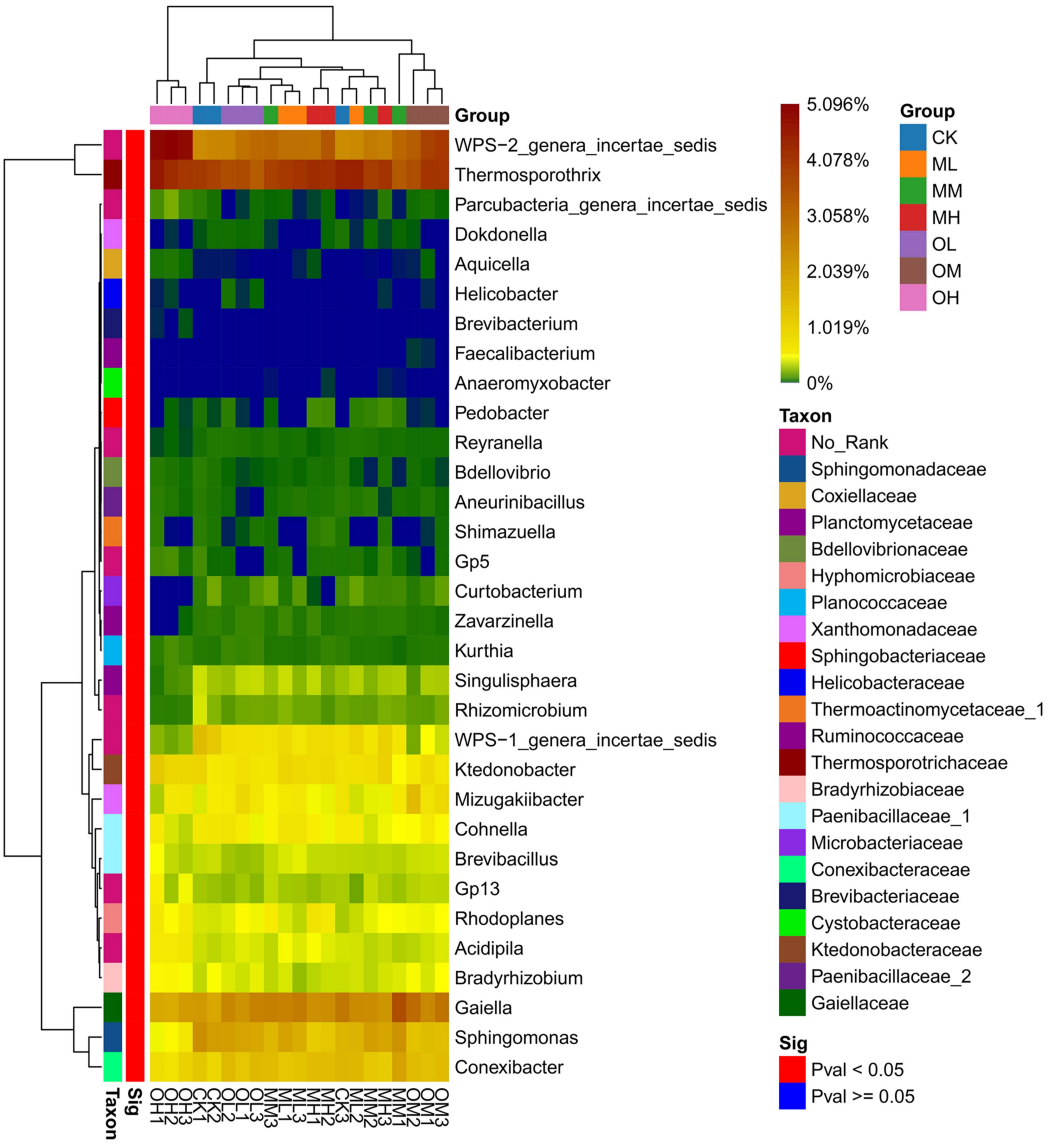
Significance of bacterial genus level in Pb-contaminated tea garden soil.

Figure 3 shows the dominant bacterial genera, determined through sequencing, including *Tumebacillus* (14–17%), *Bacillus* (11–12%), *Thermosporothrix* (8–10%), WPS-2_genera_incertae_sedis (6–12%), Nitrospira (6–8%), *Alicyclobacillus* (6–7%), *Sporosarcina* (5–7%), *Gaiella* (4–7%) and *Paenibacillus* (5–6%). The dominant bacterial genera within 5% were Gp3, Gp1, *Saccharibacteria*_genera_incertae_sedis, *Sphingomonas*, *Conexibacter*, *Mycobacterium*, WPS-1_genera_incertae_sedis and Mizugakiibacter. The abundance of *Thermosporothrix*, WPS-2_genera_incertae_sedis, *Gaiella*, *Sphingomonas*, Conexibacter and Mizugakiibacter significantly differed among the samples (*p* < 0.05; Figure 4). The abundance of *Alicyclobacillus*, *Nitrospira* and *Sporosarcina* was the highest in the soil samples treated with low Pb concentrations, whereas the abundance of Gaiella was highest in the soil samples treated with medium Pb concentrations. The abundance of *Bacillus* and *Gaiella* was higher after multistage contamination than after single instance contamination, whereas the abundance of *Thermosporothrix* and WPS-2_genera_incertae_sedis were higher after single instance contamination than after multistage contamination.

**Figure 5:**
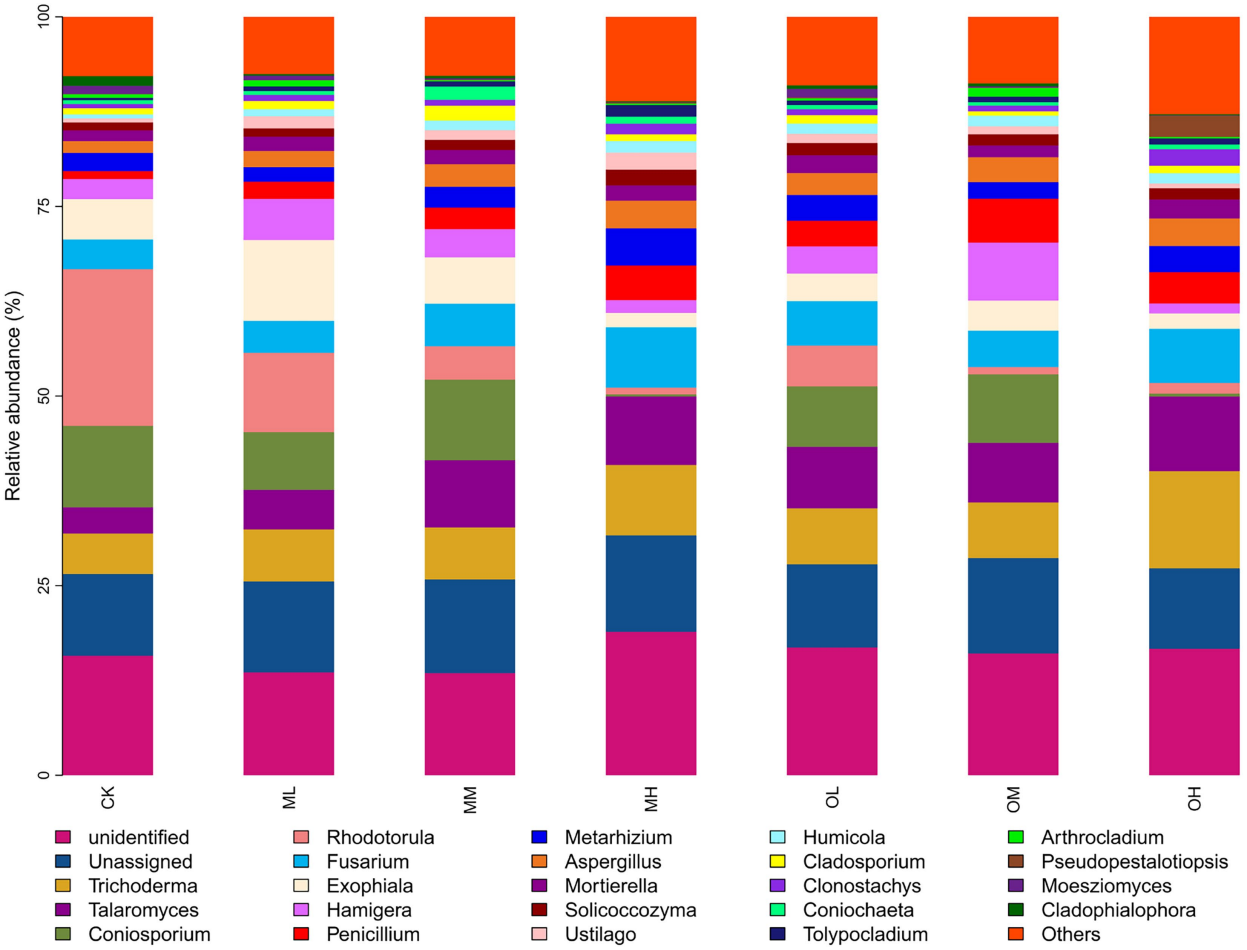
Fungal genus-level species composition in Pb-contaminated tea garden soil.

**Figure 6:**
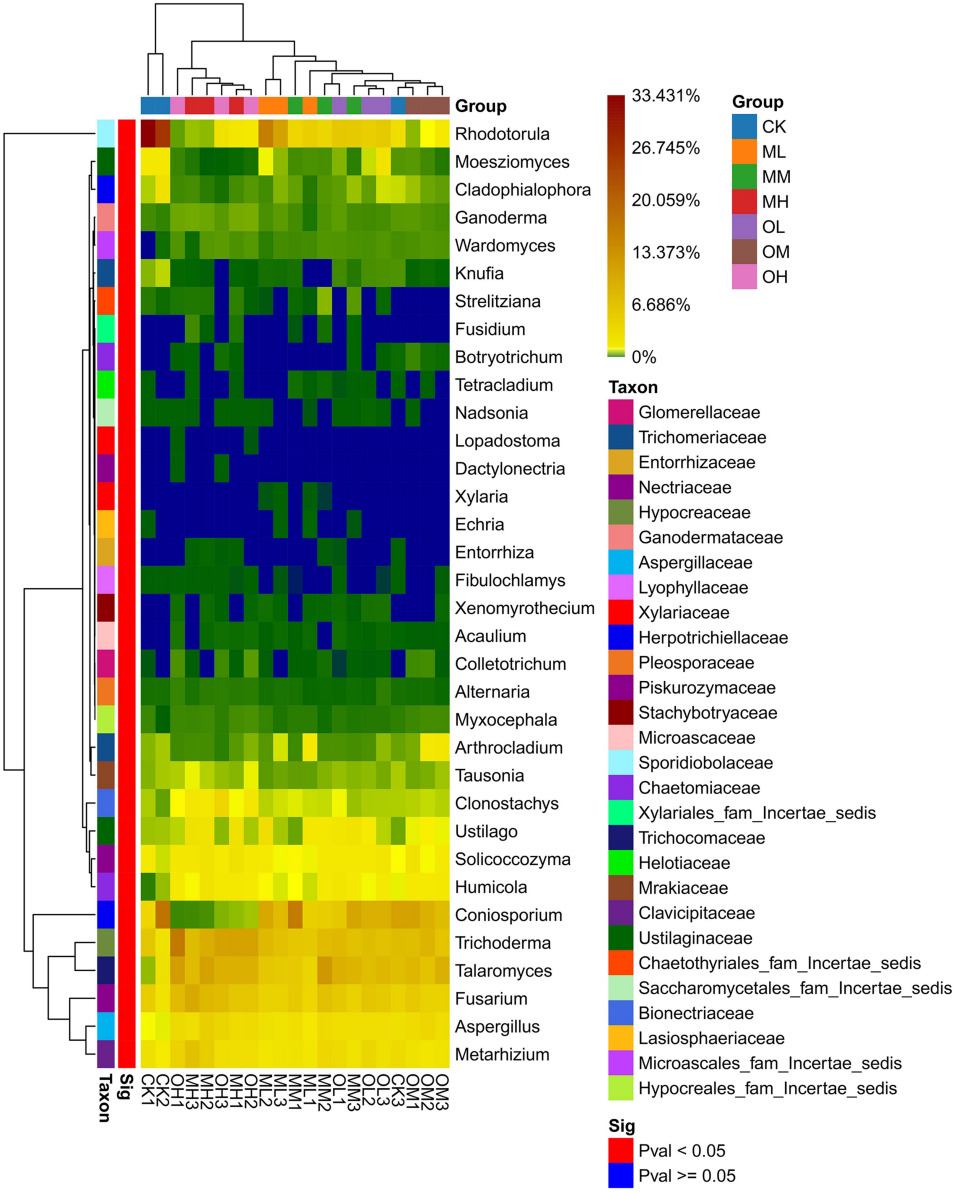
Significance of fungal genus level in Pb-contaminated tea garden soil.

Figure 5 presents the dominant fungi determined through sequencing, including *Trichoderma* (8–21%), *Talaromyces* (5–16%), *Coniosporium* (1–16%), *Rhodotorula* (2–32%), *Fusarium*, (6–14%), *Exophiala* (3–16%), *Hamigera* (2–12%), *Penicillium* (2–9%), *Metarhizium* (3–9%) and *Aspergillus* (2–6%). The dominant fungi within 5% were *Mortierella*, *Solicoccozyma*, *Ustilago*, *Humicola*, *Cladosporium*, *Clonostachys*, *Coniochaeta*, *Tolypocladium*, *Arthrocladium*, *Pseudopestalotiopsis*, *Moesziomyces* and *Cladophialophora*. The abundance of *Trichoderma*, *Talaromyces*, *Coniosporium*, *Rhodotorula*, *Fusarium*, *Metarhizium* and *Aspergillus* significantly differed among the samples (*p* < 0.05; Figure 6). The abundance of *Trichoderma*, *Talaromyces*, *Fusarium*, *Metarhizium* and *Aspergillus* was the highest in soil samples treated with high Pb concentrations, whereas the abundance of *Coniosporium*, *Exophiala* and *Hamigera* was the lowest in the soil samples treated with low Pb concentrations. The abundance of Coniosporium was the highest in the soil samples treated with medium Pb concentrations, and that of *Rhodotorula* were the highest in the soil samples treated with low Pb concentrations.

### 3.5. Redundancy analysis of microbial and environmental factors

**Figure 7:**
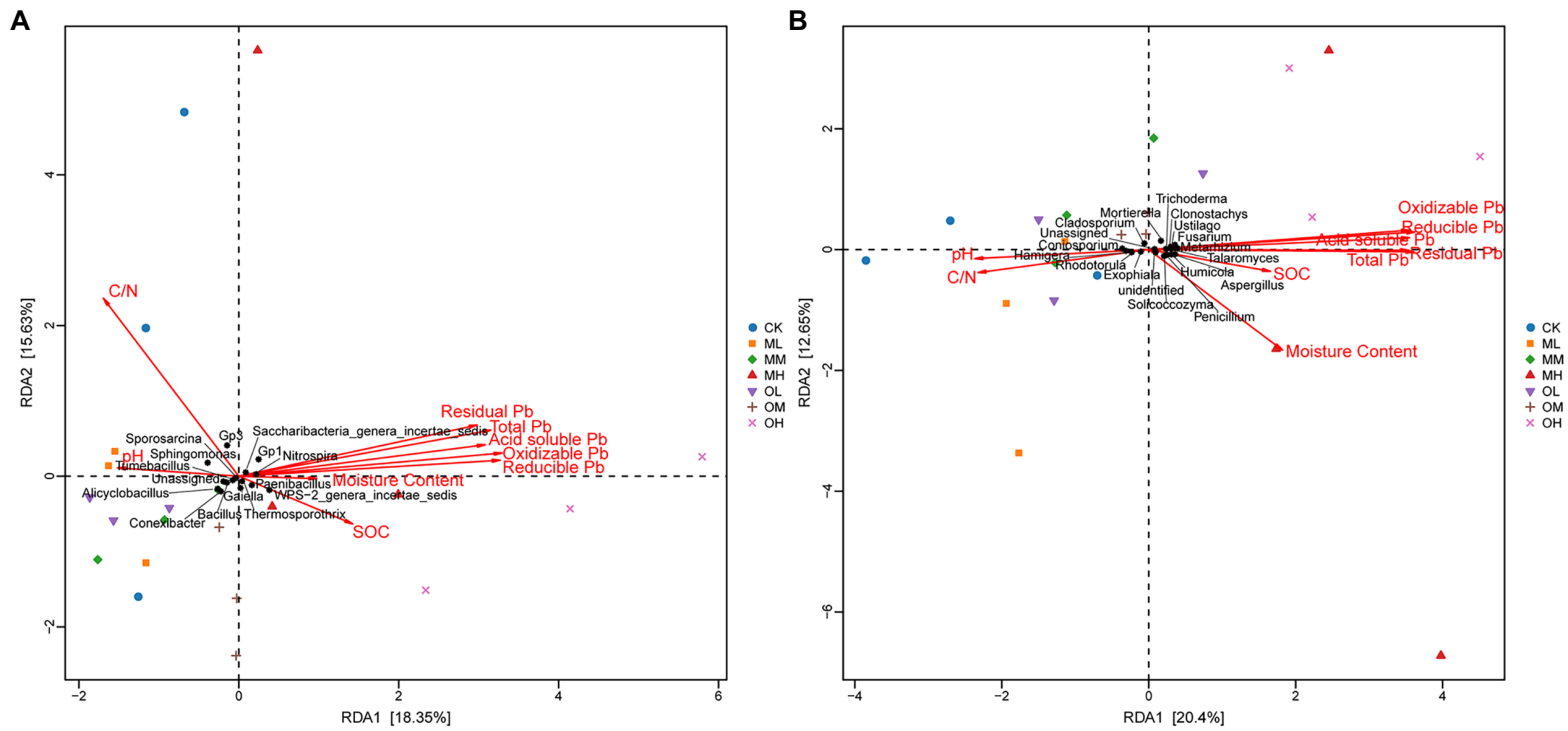
RDA of microbial genus-level abundance and environmental factors in Pb-contaminated tea garden soil. **(A)** Bacteria, **(B)** Fungi.

Figure 7A shows the results of the redundancy analysis (RDA) of bacteria at the genus level: 18.35% explanation on Axis 1 and 15.63% explanation on Axis 2. The contribution of each form of Pb to axis 1 was the largest, followed by that of organic carbon and pH, and the contribution of the soil C/N ratio to axis 2 was the largest. The pH strongly affected Tumebacillus, Nitrospira, Bacillus, Sporosarcina, Sphingomonas and Conexibacter. Organic carbon strongly affected Bacillus, *Alicyclobacillus* and *Paenibacillus* and oxidisable Pb strongly affected *Thermosporothrix*.

Figure 7B presents the results of the RDA analysis of fungi at the genus level, with 20.4% explanation degree on Axis 1 and 16.25% explanation degree on Axis 2. The contribution of each form of Pb in soil to axis 1 was the largest, followed by that of soil pH and C/N ratio, and the contribution of organic carbon to axis 2 was the largest. The residual Pb strongly affected *Trichoderma*, *Talaromyces* and *Clonostachys*, and oxidisable Pb strongly affected *Fusarium*, *Metarhizium* and *Mortierella*. The pH strongly affected *Coniosporium*, *Rhodotorula* and *Cladosporium*, the C/N ratio strongly affected *Exophiala* and *Hamigera* and organic carbon strongly affected *Penicillium*, *Aspergillus*, *Solicoccozyma*, *Ustilago* and *Humicola.*

### 3.6. Functional prediction of soil microbial community

The 4,146 differentially expressed genes annotated in the Clusters of Orthologous Genes (COG) database were classified on the basis of lineal homology to reveal the functional abundance of bacterial genes in 25 categories at the second level (Table 5). Bacterial genes involved in amino acid transport and metabolism exhibited the highest abundance (11 × 10^6^), followed by those involved in translation, ribosomal structure and biogenesis, general function, energy production and conversion, carbohydrate transport and metabolism, coenzyme transport and metabolism, transcription, cell wall/membrane/envelope biogenesis and inorganic ion transport and metabolism. The abundance of these bacterial genes in all samples treated with Pb was higher than that in the control soil and decreased as Pb concentration increased. The abundance of the bacterial genes in the soil treated with low and high Pb concentrations was higher in the single instance contamination mode than in the multistage contamination mode. The abundance of the bacterial genes in the soils treated with medium Pb concentrations was higher in the multistage contamination mode than in the single instance contamination mode.

**Table 5:** Functional notes of COG in Pb-contaminated tea garden soil bacteria.

Function class	The abundance of COG function in different treatments (×10^5^)						
	CK	ML	MM	MH	OL	OM	OH
RNA processing and modification	1	1	1	1	1	1	1
Chromatin structure and dynamics	6	6	6	6	6	6	6
Energy production and conversion	699	719	735	709	732	721	719
Cell cycle control, cell division, chromosome partitioning	151	157	157	150	158	154	155
Amino acid transport and metabolism	1,050	1,088	1,108	1,062	1,103	1,082	1,085
Nucleotide transport and metabolism	337	349	354	340	353	344	347
Carbohydrate transport and metabolism	669	695	704	680	701	684	691
Coenzyme transport and metabolism	650	673	685	657	683	669	668
Lipid transport and metabolism	503	518	534	508	531	519	511
Translation, ribosomal structure and biogenesis	907	935	947	911	947	925	931
Transcription	650	677	689	658	686	667	671
Replication, recombination and repair	431	445	452	433	451	440	442
Cell wall/membrane/envelope biogenesis	611	622	631	616	632	616	626
Cell motility	197	201	198	195	203	198	198
Posttranslational modification, protein turnover, chaperones	449	460	467	453	468	457	457
Inorganic ion transport and metabolism	487	503	512	495	511	500	503
Secondary metabolites biosynthesis, transport and catabolism	152	156	164	155	160	158	157
General function prediction only	702	723	736	710	734	715	721
Function unknown	493	507	517	495	515	497	503
Signal transduction mechanisms	419	433	437	422	438	427	429
Intracellular trafficking, secretion and vesicular transport	110	111	113	110	114	110	110
Defense mechanisms	229	236	238	230	238	231	236
Extracellular structures	1	1	1	1	1	1	1
Mobilome: prophages, transposons	35	35	36	35	36	34	35
Cytoskeleton	2	3	2	2	2	2	3

On the basis of the results of functional fungi group prediction, performed using the FUNGuild database, three fungal trophic types, namely pathotrophs, symbiotrophs and saprotrophs, were divided into seven fungal functional classes, and other undefined types are listed in Table 6. The abundance of pathotrophic–saprotrophic and pathotrophic–saprotrophic–symbiotrophic fungi significantly differed among treatments (*p* < 0.05). The abundance of pathotrophic–saprotrophic fungi was the highest in the soils (34.64–7.10%), and the highest value existed in CK treatment. The abundance of saprotrophic fungi was 10.56–19.27%, that of pathotroph–saprotrophic–symbiotrophic fungi was 5.59–13.18% and that of pathotrophic fungi was 4.11–7.66%. The abundance of undefined fungi was 39.77–61.87%. The abundance of pathotrophic–saprotrophic and pathotrophic fungi in the soil treated with Pb decreased as Pb concentration increased and was higher after multistage contamination than after single instance contamination. The abundance of pathotrophic–saprotrophic–symbiotrophic, pathotroph–symbiotrophic, saprotrophic and saprotrophic–symbiotrophic fungi increased with Pb concentration and was higher after single instance contamination than after multistage contamination. The abundance of symbiotrophic fungi decreased with an increase in Pb concentration after multistage contamination and increased with the Pb concentration after single instance contamination.

**Table 6:** Functional classification of fungi FUNGuild in Pb-contaminated tea garden soil.

Trophic Mode	Relative abundance of different treatments (%)							*p*-value
	CK	ML	MM	MH	OL	OM	OH	
Pathotroph-Saprotroph	34.64	26.70	19.88	5.51	16.23	13.95	7.10	0.002
Undefined	39.77	41.91	47.78	61.87	50.30	51.25	56.30	0.003
Pathotroph-Saprotroph-Symbiotroph	5.59	7.08	7.10	9.85	7.87	7.50	13.18	0.007
Pathotroph-Symbiotroph	0.01	0.06	0.04	0.07	0.09	0.10	0.20	0.057
Saprotroph	10.56	13.20	15.44	14.81	15.57	19.27	15.47	0.247
Pathotroph	6.94	7.66	6.10	4.66	6.54	4.75	4.11	0.306
Saprotroph-Symbiotroph	1.56	2.00	2.16	2.31	2.56	1.66	2.74	0.382
Symbiotroph	0.93	1.39	1.50	0.90	0.83	1.51	0.91	0.419

## 4. Discussion

No significant differences in the alpha index of bacteria were noted among Pb treatments (*p* > 0.05); however, the structural composition of the bacteria was affected by Pb contamination. This finding is similar to those of Diquattro et al. (2020). The enrichment and diversity indices for bacteria in the MH and OL samples were higher than those in the control sample (Table 3), and the specific OTU number for bacteria was highest in the MH samples (Figure 1). This might have been due to the use of the appropriate contamination modes and Pb concentrations, which resulted in a large number of dominant bacteria in the soil. When a small amount of Pb was added in soil, there was no inhibition on soil bacteria because Pb concentration was below the level of toxicity, and the activity and diversity of microorganisms increased. When a small amount of Pb entered the soil again after a period of time, the soil microbial activity and diversity increased. In this way, the diversity of soil microorganisms would not decline until the Pb content reached an amount that could cause serious toxicity to microorganisms and lead to their disappearance. This may, however, be due to the lower toxicity of a small amount of Pb and the adaptability of soil microorganisms over time (Ge et al., 2018; Wang et al., 2021). The NMDS analysis revealed that the difference in bacteria among samples treated with different Pb concentrations increased with Pb concentration, indicating that high Pb concentrations increased difference. Liu et al. (2018) reported that heavy-metal contamination affected microorganisms and that some microorganisms survive well or even be promoted, indicating that bacterial communities were tolerant of heavy metals to some degree. Pan and Yu (2011) showed that bacterial population size decreased significantly with an increase in heavy-metal concentration and that bacteria were more sensitive to heavy metals than were other microorganisms. Some studies have reported that some substances inhibit microbial activity in the rhizosphere of tea plants and that bacteria are more sensitive than fungi (Pandey and Palni, 1996). Because Pb is toxic to microorganisms (Beattie et al., 2018), bacteria may have decreased as Pb concentration increased in the single instance contamination mode.

In soil ecosystems, fungi can decompose organic matter, fix carbon, facilitate nutrient cycling, and improve soil structure; the diversity of fungi determines the diversity of soil ecosystems and plant productivity (Frąc et al., 2018). Fungal abundance and diversity were the highest in the soil samples treated with high Pb concentrations not only after multistage contamination but also after single instance contamination, Pb contamination significantly increased fungal diversity (*p* < 0.05; Table 4). Diquattro et al. (2020) reported that the high concentration of antimony significantly increased the number of culturable heterotrophic fungi in sandy clay loam (*p* < 0.05). Zhang et al. (2022) also reported that medium-concentration Pb treatment (500 mg kg^−1^) was more conducive to maintaining higher activity and diversity of fungi in the cinnamon soil, and there was a significant inhibitory effect on the number and diversity of fungi when the Pb content in soil reached 2,500 mg kg^−1^(*p* < 0.05). The NMDS analysis indicated that fungi differed between the soil samples treated with high Pb concentrations and those treated with low and medium Pb concentrations. Fungi exhibited a high tolerance (in terms of cell viability) to heavy-metal toxicity, probably because they have thicker cell walls (Xie et al., 2017). Another explanation is that fungi have a symbiotic relationship with plants, making them more resistant to changes in environmental factors (Deng et al., 2018). Therefore, the soil treated with Pb had the higher fungal abundance and diversity regardless of multistage pollution mode or single instance pollution mode.

Soil microorganisms adapt to long-term heavy-metal contamination through changes in microbial community composition and structure rather than in diversity and uniformity (Li et al., 2017). Cimermanova et al. (2021) investigated the effects of heavy metals on actinomycetes and observed that actinomycetes were highly tolerant to Pb. In this study, Thermosporothrix and Gaiella with significant differences between different Pb treatments were belonged to Actinomyces (*p* < 0.05). Liu et al. (2013) proposed that planting plants in soils contaminated by heavy metals could increase the complexity of the actinomycete community; this may be the reason for the dominance of actinomycetes in this study. RDA revealed that oxidisable Pb was responsible for the abundance of Thermosporothrix among the treatments. RDA also revealed that Pb contamination had stronger effects on fungal dominant genera than on bacteria. Residual Pb was responsible for the significant difference in the abundance of Trichoderma and Talaromyces among Pb treatments, whereas oxidized Pb was responsible for the significant difference in the abundance of Fusarium and Metarhizium among Pb treatments (*p* < 0.05). The dominant fungal genus in tea garden soil has a strong response to Pb contamination. Chen et al. (2014) reported that heavy-metal contamination considerably affects the fungal community structure. In addition, the bacterial community is not only dependent on heavy metals but is also affected by pH, nutrients, and organic matter content (Lauber et al., 2008; Ma et al., 2020). This study determined the dominant bacterial genera Tumebacillus, Nitrospira and Sporosarcina to be susceptible to the effects of soil pH, whereas Bacillus and Alicyclobacillus were susceptible to the effects of organic matter.

By performing PICRUSt2 function prediction, we annotated bacterial function in the COG second-level function and linked microbial changes to biological function through a comparison with the database. The results indicated that amino acid transport and metabolism, carbohydrate transport and metabolism, coenzyme transport and metabolism and inorganic ion transport and metabolism were the main functions of bacteria in the one-time functional layer (Sun et al., 2019). Translation, ribosomal structure and biogenesis, energy production and conversion and transcription were genetic information and cellular processes observed in the one-time functional layer (Sun et al., 2019). These results indicated that Pb contamination enhanced metabolic function, genetic information processing and the cellular processes of dominant bacteria in tea garden soil; increases in Pb concentration negatively affected the dominant functions of bacteria, indicating that the dominant bacteria in the soil were resistant to Pb. The dominant functions of soil bacteria in the single instance contamination mode were generally stronger than those in the multistage contamination mode. Some studies have indicated that the number of tolerant microorganisms in sites contaminated by heavy metals increased with the concentration of heavy metals (Xie et al., 2016). This might be the reason that the dominant function of bacteria in the single instance contamination mode was stronger than that in the multistage contamination mode. Another reason might be that contaminated microbial communities require more carbon sources to obtain energy for biochemical functions than do those in uncontaminated soils (Giller et al., 1998).

FUNGuild functional predictive analysis was performed to classify fungi by nutritional type and pattern. The relative abundance of pathotrophic–saprotrophic, saprotrophic, pathotrophic–saprotrophic–symbiotrophic and pathotrophic fungi was high, whereas the relative abundance of symbiotrophic fungi was low. Some studies have indicated that the functional transformation of pathotrophic–saprotrophic–symbiotrophic fungi was susceptible to the effects of human activity (Chen et al., 2019). Pathotrophic fungi obtain nutrition mainly from within host cells; thus, they are harmful to plant growth (Xiao et al., 2021). Endophytic microorganisms (including bacteria and fungi) were likely to closely interact with their hosts and were protected from adverse changes in the environment (Deng and Cao, 2017). In this study, the abundance of pathotrophic–saprotrophic and pathotrophic fungi decreased with an increase in the Pb concentration, whereas the abundance of symbiotrophic fungi increased with an increase in the Pb concentration, indicating that although Pb contamination reduced the number of fungal communities, it improved the health of soil fungal communities and that the composition was more favorable in the single instance contamination mode than in the multistage contamination mode. Endophytic fungi could establish a special symbiotic relationship with plants to create plant immunity, promote plant growth and form specific metabolites (Han et al., 2021). This might be the reason that the fungi in the soil resisted high Pb concentrations. The functional prediction of FUNGuild is based on the literature and data analysis and can only be used to interpret fungal function within a certain capacity (Wang et al., 2018). In this study, 56.30% of fungal functions were not successfully interpreted, and the function of complex fungal communities in soil requires further study.

## 5. Conclusion

The abundance and diversity of bacteria varied considerably between different Pb contamination modes and were higher in the multistage contamination mode. In both contamination modes, the abundance and diversity of fungi were the highest in the soil samples treated with high Pb concentrations. The composition of the fungal community was more affected than that of bacteria by Pb contamination. Fungal dominant genera were highly susceptible to residual and oxidized Pb, and few dominant genera in the bacterial community were affected by oxidisable Pb. The predicted main function of the bacterial community was amino acid transport and metabolism, and the trophic mode of the fungal community was mainly pathotroph–saprotroph. The effects of the multistage contamination treatment of Pb on soil microorganisms were stronger and reflected environmental risk more than the single instance contamination treatment did.

## Data availability statement

The datasets presented in this study can be found in online repositories. The names of the repository/repositories and accession number(s) can be found below: BioProject, PRJNA934694 and PRJNA934753.

## Funding

This work was supported by the National Natural Science Foundation of China (41401278), and the Natural Science Foundation of Anhui province of China (2008085MC97).

## Author contributions

ZZ: conceptualization, formal analysis, and writing original draft. QD: formal analysis and methodology. HY: formal analysis and validation. GG: designing the experiments and revising the manuscript. All authors contributed to the article and approved the submitted version.

## Conflict of interest

The authors declare that the research was conducted in the absence of any commercial or financial relationships that could be construed as a potential conflict of interest.

## Publisher’s note

All claims expressed in this article are solely those of the authors and do not necessarily represent those of their affiliated organizations, or those of the publisher, the editors and the reviewers. Any product that may be evaluated in this article, or claim that may be made by its manufacturer, is not guaranteed or endorsed by the publisher.
